# In A Class of Their Own: Multiclass Random Forests Reveal Distinct Habitat Needs of Fledglings Relative to Adults in Two Grassland Birds

**DOI:** 10.1002/ece3.74218

**Published:** 2026-08-19

**Authors:** Jacy S. Bernath‐Plaisted, Nicole A. Sanders, Katharine J. Ruskin, Brian J. Olsen, Arvind O. Panjabi, Maureen D. Correll

**Affiliations:** ^1^ Bird Conservancy of the Rockies Fort Collins Colorado USA; ^2^ Ecology and Environmental Sciences Program University of Maine Orono Maine USA; ^3^ School of Biology & Ecology University of Maine Orono Maine USA; ^4^ U.S. Fish & Wildlife Service Hadley Massachusetts USA

**Keywords:** Baird's sparrow, Grasshopper sparrow, grassland birds, machine learning, multiclass, nest‐site selection, ontogeny, post‐fledging habitat

## Abstract

Habitat needs often differ among adult and juvenile life‐history stages. Such differences not only reveal important aspects of basic ecology, but may also have implications for conservation and management of species. Ontogenetic habitat selection is an inherently multiclass challenge, meaning that it requires modeling more than two classes of use location, but there are few examples of resource‐selection functions (RSF) that directly compare both adult and juvenile habitat use with random locations and one another. We developed multiclass Random Forests, a type of machine learning model with high predictive accuracy, to model habitat selection by fledglings and nesting adults of two declining North American grassland bird species, Baird's Sparrow and Grasshopper Sparrow, in an integrated framework. We find that fledglings of both species exhibit habitat use that is distinct from both random locations and conspecific nest sites. Habitat use by fledgling Grasshopper Sparrow was consistent with existing work documenting the importance of vegetation cover during this vulnerable period. However, Baird's Sparrow fledglings displayed unexpected preferences for microhabitat diversity and open structure, possibly mediated by the species' sensitivity to exotic vegetation and shifts in habitat preference with fledgling age. Our work highlights differing habitat needs during the post‐fledging phase for two species of concern, documents the potential of species traits and sensitivities to affect habitat use during a vulnerable life‐history stage, and advances an underutilized analytic approach that may enhance the functionality of RSF to answer multiclass questions in ecology.

## Graphical abstract

Adults and juveniles across taxa often have unique habitat needs as a result of differing selective pressures during these periods, and such differences may inform the conservation and management of species. Here, we apply a novel multiclass analytic approach to compare the habitat use of nesting adults and fledglings in two declining grassland bird species. We find that adults and fledglings display distinct preferences from one another and random locations, and that fledglings may be more vulnerable to some habitat threats, such as invasive species.

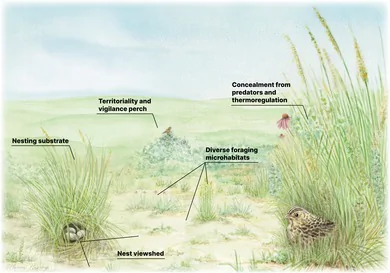

## 1 Introduction

Habitat loss remains the primary driver of global biodiversity declines (Jaureguiberry et al. 2022). Understanding the habitat requirements of species is an important goal of ecological studies, and instrumental to informing conservation efforts (e.g., Indermaur and Schmidt 2011; Kramer et al. 2021). However, habitat selection may differ across life‐history stages with implications for the ecology and conservation of species (e.g., Desbiez et al. 2020; Groff et al. 2017). Studies of habitat selection across ontogeny often reveal unique aspects of species' ecology and identify vulnerable life‐history phases (e.g., Hannon and Martin 2006; Thorsen et al. 2022).

Juvenile or immature life‐history stages are often underrepresented in habitat studies across taxa (e.g., Ciotti et al. 2025; Jones et al. 2025). In avian species, studies of habitat selection most often focus on the broad associations of adult birds, (e.g., Guo et al. 2023; Lipsey and Naugle 2017; Spiller and King 2021), or their nest‐site preferences (e.g., Chalfoun and Schmidt 2012; Martin 2001). This likely stems from the greater visibility of adults in many species, and the difficulty involved in locating and tracking the movements of juveniles—something that has only become possible for passerines with the advent of smaller radio tags in the last several decades (Anders et al. 1997; Thomson et al. 1999). Yet, intrinsic growth rates of species are often sensitive to juvenile recruitment (Anders and Marshall 2005; Cox et al. 2014), making the study of post‐fledging habitat needs important both to advancing fundamental ecology and informing conservation practices (Fiss et al. 2021; Hannon and Martin 2006). In recent decades, general knowledge and synthesis around the post‐fledging phase have substantially increased (Cox et al. 2014; Jones et al. 2025; Russell 2001). However, the habitat needs and behavior of fledglings for many species remain poorly studied or entirely undescribed, and microhabitat use by fledglings is likely to diverge from that of adults given differing selective pressures during these distinct periods. For example, adults must balance their own survival and lifetime reproductive success with the success of each nesting effort while fledglings face immediate selective pressure simply to survive (Chalfoun and Schmidt 2012; Jones et al. 2025).

**FIGURE 1:**
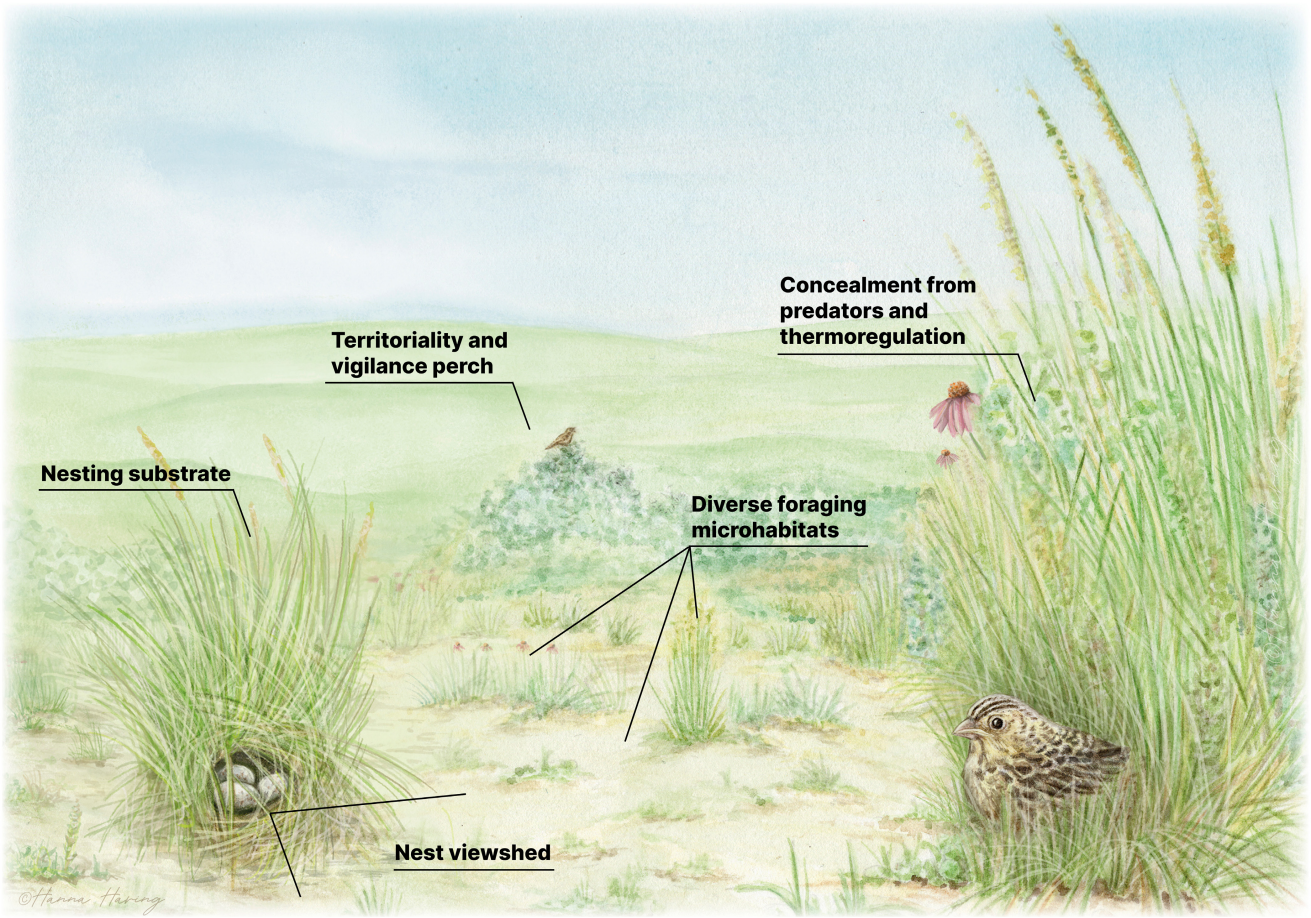
An idealized depiction of how microhabitat elements could be important for fledglings and nesting adults. Illustration by H. Haring.

Nest‐site selection is a critical adult choice with fitness consequences for survival and reproductive success (e.g., Brussee et al. 2023; Martin et al. 2015), and many selective pressures may influence habitat preferences during this period (Figure 1). Habitat selection in birds can occur at landscape, territory, and nest site scales (typically 1–5 m; Jedlikowski et al. 2016), but often microhabitat at the nest site has the greatest impact on nesting success (Shew et al. 2019). For example, dense vegetation directly at nest sites provides concealment from predators (e.g., Segura et al. 2012). At the same time, open areas may be important for nest site viewsheds that increase survival of adults while incubating and brooding (Dorsey et al. 2025), and offer foraging opportunity in bare patches (Tagmann‐Ioset et al. 2012). By contrast, the post‐fledging period is characterized by low survival that strongly increases with age and mobility (Jones et al. 2025), and during the first 2–4 weeks after leaving the nest (typically 10–20 days in the northern hemisphere; Russell 2001) fledglings are still fed by parents. Thus, avoiding mortality is likely the strongest selective pressure during this phase (Marchetti and Price 1989), and one that may drive fledglings to seek denser vegetation where they can find concealment from predators and buffering from climate exposure (Figure 1; Jones et al. 2025; Jones and Bock 2005; Vitz and Rodewald 2011).

Relatively few studies directly compare habitat characteristics among locations selected by fledglings, nesting adults, and random locations, and those that do typically achieve this through comparison of multiple binary classification modeling efforts (e.g., Fiss et al. 2021; Raybuck et al. 2020). However, many processes in ecology are not inherently binary (Bourel and Segura 2018), and the development of multiclass analytic methods offers a more integrated approach to directly compare characteristics of fledgling habitat use, nest sites, and random locations. Such an approach would likely reduce propagation of error that could occur through comparison of multiple separately estimated statistics and simplify the modeling process. Perhaps more importantly, multiclass models could make interpretation and comparison of class use probabilities and feature importance more powerful and intuitive by allowing side‐by‐side comparison and summary of class characteristics. Multiclass methods are well aligned with recent developments in the application of machine learning (ML) to ecological classification (Pichler and Hartig 2023). Ensemble modeling approaches such as Random Forest (RF) are among the most powerful models available for classification and are increasingly preferred over classical regression approaches for their high predictive accuracy (e.g., He et al. 2018). RF is a type of decision tree model that averages classifications over numerous tree iterations to produce unbiased and accurate predictions (Breiman 2001), and these models have been shown to handle multiclass problems better than other approaches (Bourel and Segura 2018; Pichler and Hartig 2023; Sidumo et al. 2022). Additionally, RF is well suited to resource selection functions (RSF) in ecology, which often involve high‐dimensional data, multicollinearity in environmental variables (e.g., compositional vegetation data), and the need for unbiased prediction—a property that may be compromised by trimming of predictor sets to avoid correlation (Hanberry 2024). Despite these desirable characteristics, ML has seen little application in RSFs until recently (Griffin et al. 2021), and multiclass methods are seldom employed in ecological studies (Sidumo et al. 2022).

We develop multiclass RF models to directly compare habitat selection during the post‐fledging and nesting phase in two North American grassland birds of conservation interest, Baird's Sparrow (*Centronyx bairdii*)—for which fledgling habitat use has not been formally described—and Grasshopper Sparrow ( *Ammodramus savannarum* ). Grassland birds are among the most steeply declining avian groups in North America (Rosenberg et al. 2019), primarily as a consequence of historical and ongoing grassland habitat loss (Augustine et al. 2021; Lark 2020). Thus, they are frequently the target of conservation and habitat management efforts (Bernath‐Plaisted, Correll, et al. 2023). Both Baird's Sparrow and Grasshopper Sparrow nest in the mixed‐grass prairie of the Northern Great Plains (NGP) where their breeding distributions overlap, though Grasshopper Sparrow is more widely distributed (Figure S1). The two species share similar natural histories and both are cryptic ground nesters that require open grassland habitat and avoid high shrub densities (Green et al. 2020; Vickery 2020). In the NGP, both species are associated with taller and denser vegetation structure maintained by moderate to low grazing disturbance (Lipsey and Naugle 2017).

Our study had three objectives: (1) understand post‐fledging habitat needs in two declining grassland birds and inform their management in the NGP, (2) contribute to existing knowledge highlighting the ecological importance of ontogenetic habitat selection, and (3) advance a rarely employed analytic approach to RSF by developing multiclass RF models to directly compare use probability of fledglings, nest sites, and random locations. We located and monitored nests of both species, and tracked fledglings by radio telemetry. At fledgling use locations, nest sites, and random locations, we collected data describing vegetation structure and primary cover classes. We supplemented these data with high‐resolution spatial observations collected by Unpiloted Aerial Systems (UAS) to characterize primary productivity (Green et al. 2019) and topography (Frey et al. 2008). We hypothesized that if post‐fledging habitat use is most strongly driven by high mortality during this vulnerable phase, then fledglings would show directional selection for greater vegetation height and density. By contrast, if nest sites selected by adults are influenced by multiple selective pressures, and a need to exploit diverse resources at the nest‐site scale, we predicted that nest sites would display greater habitat diversity, and greater presence of open habitat to support adult foraging and vigilance. Our study seeks to inform the habitat needs and management of two sensitive species, and simultaneously develop an analytic approach that could have broader application to ontogenetic habitat studies, and other multiclass problems in ecology.

## 2 Materials and Methods

### 2.1 Study Area

Our study was part of a regional grassland bird demography conducted in the mixed‐grass prairie of the NGP. Additional details on this research and study area may be found in Bernath‐Plaisted et al. (2021). We collected fledgling and nesting habitat data at two study sites (Figure S1), one located in Valley County, Montana, USA (48°39′ N, 106°28′ W) and one in Golden Valley County, North Dakota, USA (46°37′ N, 103°59′ W). Each study site consisted of two mixed‐grass prairie pastures ranging in size from 128 to 177 ha, and pastures were periodically grazed throughout the study duration. Our Montana site included one privately owned parcel and an adjacent property managed by the Bureau of Land Management (BLM) and leased for public grazing. In North Dakota, both study pastures were located on the Little Missouri National Grassland under the jurisdiction of the USDA Forest Service (USFS) and grazed by lessees. Both study sites occurred at ~900 m of elevation, and were situated in large grassland contexts. Our sites were characterized by flat to gently rolling topography, natural drainages and seasonal wetlands, and low woody‐plant density. The vegetation community was composed of native warm and cool season mixed‐grass prairie grasses, as well as diverse native forbs. There was a significant presence of invasive cool season grasses at both sites including Crested Wheatgrass ( *Agropyron cristatum* ), Kentucky Bluegrass ( *Poa pratensis* ), and Smooth Brome ( *Bromus inermis* ). Broadly, habitat conditions were similar at Montana and North Dakota study sites, through the former had greater cover of invasive (Figure S2).

### 2.2 Nest Location and Fledgling Tracking

From late May to early August, 2018, we located and monitored nests of Baird's Sparrow and Grasshopper Sparrow, and subsequently tagged and tracked fledglings of both species by radio telemetry. We located nests using rope dragging (Winter et al. 2003), behavioral observation (Rodewald 2004), and incidental discovery while traversing study plots. We followed common practices employed in nest monitoring studies of grassland birds, and refrained from searching in cold (< 10°C) and wet conditions (active precipitation or excessive dew), marked nests discretely with flagging, and returned to nests at regular intervals (2–5 days) to monitor contents (Bernath‐Plaisted et al. 2021; Jones et al. 2010).

We monitored all active nests, and visited when nestlings were ~7 days to band and outfit nestlings with transmitters. Both Baird's and Grasshopper Sparrow typically fledge at ~9–10 days (Jones et al. 2010). During tagging visits, we banded nestlings with a United States Geological Survey (USGS) federal aluminum band. We weighed nestlings to determine if they weighed > 11 g such that transmitters were no more than 3% of body mass. Of this subset, we randomly selected 1–2 individuals (depending on the brood size and availability of transmitters) to outfit with a VHF radio transmitter (PicoPip Ag337; 0.29 g, ~20–30‐day battery‐life; Lotek Wireless, Seattle, WA, USA) using a leg‐loop harness (Rappole and Tipton 1991). We tracked birds daily using a receiver (Biotrack VHF Receiver; Lotek Wireless) and Yagi antenna (Advanced Telemetry Systems, Isanti, MN, USA), and recorded fledgling locations with a GPS unit (etrex 10 and etrex 20; Garmin Ltd., Olathe, KS, USA). During tracking, we exercised caution when approaching fledgling locations to avoid trampling birds with limited flight ability, or influencing marked locations. We tracked fledglings until a mortality was confirmed, the transmitter battery died, or the bird was presumed to have departed the study site (e.g., older fledglings towards the end of the season). When birds could not be detected, we walked the perimeter of study pastures with a Yagi antenna to confirm the transmitter was truly absent from the study area (Bernath‐Plaisted et al. 2021).

### 2.3 Habitat Data Collection

At fledgling locations, we collected eight ground measurements within a 5‐m radius. These included average vegetation height (cm), defined as the height at which 80% of biomass was visually estimated (Fisher and Davis 2010), and cover estimates of the following cover classes (%): bare ground (including rock and lichen cover), dead grass (vertical and still rooted in soil), forb (herbaceous non‐graminoid plants), litter (dead, horizontal plant matter and other detritus), live grass, shrub, and non‐native species with all covers but this last category summing to 100. For each fledgling use location, we also collected vegetation data at a random location within the same study pasture. Random locations were selected from a list of pre‐generated random bearings and distance combinations applied from the location the bird was tracked to. To ensure that our random sample captured vegetation conditions that were realistically available (Northrup et al. 2013), our random distances were confined to less than 50 m and less than 100 m, respectively, for younger (1–10 days) and older (> 10 days) fledglings. These distance thresholds were based on analysis of daily fledgling movements from previous years of the study (Bernath‐Plaisted et al. 2021). We refrained from collecting vegetation data the same day that tracking occurred to avoid disturbing or trampling fledglings, but rather returned 2 days later to perform vegetation surveys at fledgling locations and associated random points. We did not collect vegetation data at mortality locations as these may not have reflected habitat selection by fledglings if predators had moved remains. At each nest location and one paired random location within the same pasture, generated in ArcMap 10.6 (ESRI, Redlands, CA), we collected the same vegetation metrics. We could not gather vegetation data while nests were active, but we did so within 3 days of termination to ensure surveys captured vegetation that resembled conditions while the nest was active.

We also gathered high‐resolution imagery characterizing primary productivity and topographic conditions to supplement ground measurements. We piloted an eBee Plus fixed‐wing drone (senseFly, Switzerland) equipped with multispectral cameras to collect spectral reflectance data. We collected data three times during the breeding season at each study site (approximately every 30 days) from mid‐May through early August to capture phenological changes in the habitat (Bernath‐Plaisted, Ribic, et al. 2023). We used these data to generate a digital elevation model (DEM) (e.g., Kršák et al. 2016), and to calculate the normalized difference vegetation index (NDVI) using the formula [NIR − red]/[NIR + red] (e.g., Ghazal et al. 2015). Both products were rendered at resolutions of < 15 cm. We extracted mean values for each location (fledgling use, nest site, and random points) by creating a 5‐m radius buffer around each point using ArcMap. For NDVI, we extracted imagery from the flight closest to the survey date. Additional details describing UAS flights, camera specifications, and photogrammetry may be found in Text S1.

Finally, we used the measurements previously described to derive three additional variables characterizing habitat at use and random locations (Text S1). These included an “open” habitat variable combining bare ground and litter cover, an index of cover‐class diversity calculated using inverse Simpson's Diversity Index (Pitkänen 1998), and an elevation anomaly variable from UAS data that corrected for differing elevations among study pastures by redefining elevation as the difference from mean site elevation.

### 2.4 Data Preparation

We collated fledgling use locations, nest sites, and random locations into a single dataset for each species (Table S1). Next, we used Synthetic Minority Over‐sampling Technique (SMOTE) (Chawla et al. 2002) implemented in the *themis* package (Hvitfeldt 2025) to address class imbalance in our data (e.g., differing numbers of fledgling, nest site, and random locations; Table S1) (Anusha et al. 2023). We performed SMOTE only on the training data after partitioning holdout/test data (70–30 for Baird's Sparrow, 65–35 for Grasshopper Sparrow; Table S2; Text S1) such that data reserved to evaluate model performance contained only observations never seen by the learner and not up‐sampled during SMOTE.

### 2.5 Multiclass Random Forests

To directly compare the influence of habitat variables on use probabilities between fledgling locations, nest sites, and random points, we developed multiclass Random Forest (RF) (Breiman 2001) classifiers for each species in an R coding environment (R Core Team 2026). We constructed multiclass RF separately for both Baird's Sparrow and Grasshopper Sparrow using the *ranger* package (Wright and Ziegler 2017) to train models such that a three‐level factor response describing habitat use (0 = random location, 1 = fledgling use, 2 = nest site) was predicted by 9 habitat covariates at a 5‐m scale (Table 1): dead grass cover (%), diversity (inverse Simpson's index, effectively the number of cover types), elevation anomaly (m), exotic cover (%), forb cover (%), NDVI (−1 to 1 index of productivity), open cover (%), shrub cover (%), and vegetation height (cm). We did not include bare ground cover (%) or litter cover (%) as these were combined into an open cover variable (Text S1). We also did not include live grass cover (%), as this contained similar information to NDVI, and was the strongest source of correlation in our data (Figure S3). Although RF can model correlated predictors, a covariate with multiple strong correlations could still limit inference and interpretation of partial dependence (Breiman 2001; Molnar 2022). Additional details describing model tuning and hyperparameters are available in Table S2 and Text S1.

**TABLE 1:** Covariates modeled in multiclass Random Forests classifying habitat use by fledgling and nesting adult Baird's Sparrow and Grasshopper Sparrow.

Covariate	Type	Collection method	Reason
Dead grass cover	Measured	Manual	May inform cover availability for nest sites, common grassland measurement
Diversity	Derived	Manual	May capture resources available to birds at different life‐history stages
Elevation anomaly	Derived	UAS	Topography may influence bird habitat selection as well as cattle grazing
Exotic cover	Measured	Manual	Some grassland bird species are sensitive to non‐native cover
Forb cover	Measured	Manual	Forbs with broader leaves and flowers could provide cover and food
Vegetation height	Measured	Manual	Tall vegetation likely provides concealment from predators and thermoregulatory benefits
NDVI	Measured	UAS	Primary productivity may indicate dense vegetation cover and food
Open cover	Derived	Manual	May expose fledglings and nests to predators, but also could provide foraging opportunity and viewshed
Shrub cover	Measured	Manual	High‐density shrubs are typically avoided by grassland birds, but low‐density may provide cover, microclimate, and perch availability

We evaluated model performance using confusion matrices to classify observations in test data, and by calculating Area Under the Curve (AUC) (Ling et al. 2003) using the *pROC* package (Robin et al. 2011). To interpret model output, we extracted both global and local feature importance (Kopitar et al. 2019). This allowed us to understand which variables most influenced overall classification performance, but also how their importance differed among identification of specific classes (e.g., fledging use or nest site). Finally, to understand the directionality of relationships, we calculated partial dependence using the *pdp* package (Greenwell 2017).

### 2.6 Age‐Habitat Regression

To further assess evidence of ontogenetic habitat preferences, and because fledgling age would have been difficult to integrate into our classification models (e.g., a factor with many levels or a continuous variable that would implicitly model interaction but offer little inference), we used simple linear‐regression to examine shifting habitat use with fledgling age. For all habitat variables, we summarized values at use locations by fledgling age for each species, 1–20 days (days post‐fledge). We limited our regression to this range because we had little replication at greater ages (Figure S5). We then performed univariate linear regression with age as the independent variable and habitat variables as the dependent response, and evaluated the significance of the relationship (*p <* 0.05) and goodness of fit (*R* ^*2*^).

## 3 Results

### 3.1 Summary Data and Fledgling Movements

During the summer of 2018, we located nests of Baird's Sparrow (*n* = 45) and Grasshopper Sparrow (*n* = 75), and tracked the movements of fledglings of both species by radio telemetry (*n* = 43 and *n* = 31, respectively). We collected habitat data at all nest locations for both species, as well as 170 and 210 fledgling use locations, respectively. We gathered habitat data at a combined 504 random locations (Table S1). Summaries of habitat data by class for all variables are available in Table S3 and Figure S4. We tracked fledgling Baird's Sparrow and Grasshopper Sparrow at ages ranging from 1 to 27 days (days post‐fledge; Figure S5). Daily fledging movement distances generally increased with age for both species in our study (Bernath‐Plaisted et al. 2021), and fledglings moved a mean distance of 35 m from the previous day's location prior to developing strong flight (1–10 days), and with standard deviations of 26.7 and 32.2 m, respectively for the two species. At ages of > 10 days, both species moved further and with greater variation; Baird's Sparrow averaged 91.5 m per day (SD = 94.9) and Grasshopper Sparrow 80.9 m (SD = 67.2).

### 3.2 Habitat Selection by Fledglings and Nesting Adults

**FIGURE 2:**
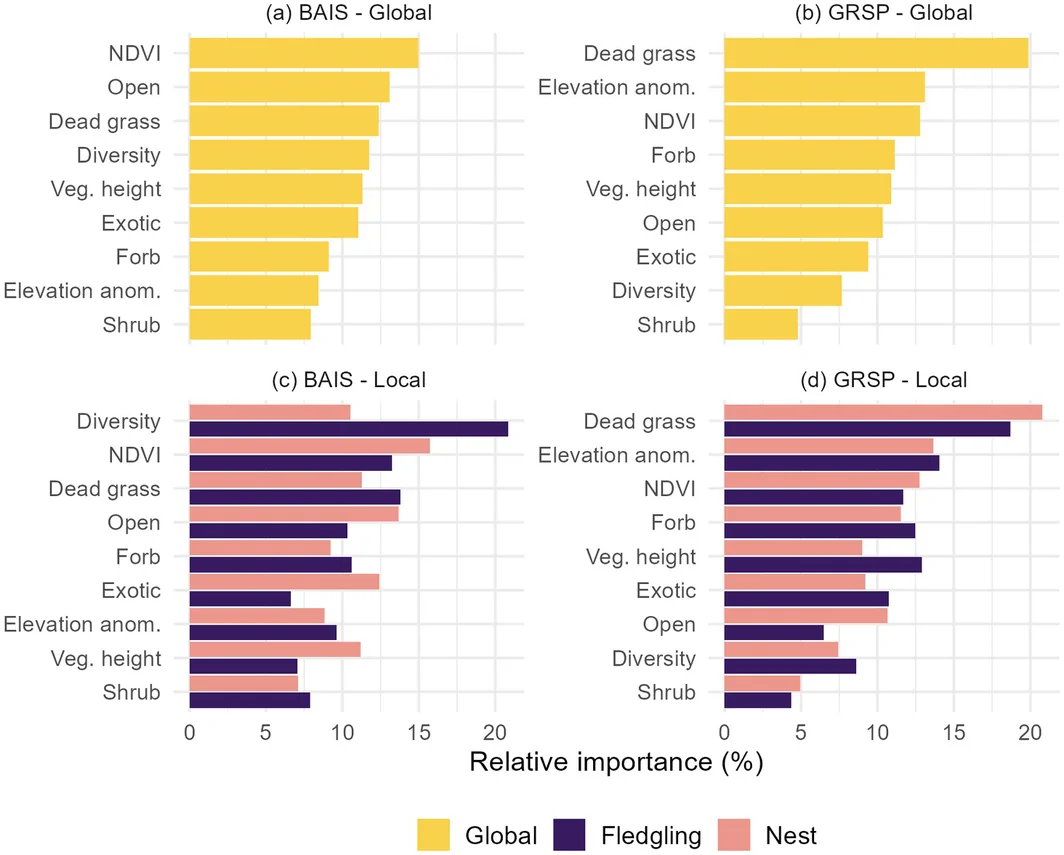
Feature importance for habitat characteristics in multiclass Random Forests predicting use by fledglings and nesting adults for Baird's Sparrow (BAIS) and Grasshopper Sparrow (GRSP) expressed as a percentage. Global importance describes overall impact of features on model performance (a, b), while local importance describes the influence of variables in distinguishing specific classes (c, d).

We evaluated multiclass RF models comparing use probability among fledglings, nest sites, and random locations using confusion matrices with test data only. The Baird's Sparrow classifier performed with greater accuracy (balanced accuracy = 70%; AUC = 0.73) than the Grasshopper Sparrow model (balanced accuracy = 63%; AUC = 0.70). Models of both species had high specificity (e.g., fledgling use and nests locations were rarely misclassified), but lower sensitivity (e.g., not all use points and nests were detected), suggesting while there was a distinct signature of microhabitat use for both classes, use locations were not always well distinguished by the variables we modeled (Table S4). Feature importance revealed that global model performance in both species was driven strongly by dead grass cover, primary productivity (NDVI), and vegetation height (Figure 2a,b). Additionally, Baird's Sparrow responded to open cover, cover diversity and exotic cover while Grasshopper Sparrow was more sensitive to elevation and forb cover. (Figure 2a,b). However, importance of features in distinguishing fledgling locations and nest sites selected by adults varied from global importance, and differed among species (Figure 2c,d). For both species, forb cover was more important to fledgling use probability than nest sites while open cover was more important to identifying the latter (Figure 2c,d). In Baird's Sparrow, cover diversity was notably more influential to fledgling use while exotic cover had greater importance to nest sites (Figure 2c,d). In Grasshopper Sparrow, dead grass cover had more influence on nest‐site selection than fledgling use, and vegetation height was more important to fledging use (Figure 2c,d). Critically, these relationships reflect the importance of variables in distinguishing classes, but not the directionality of relationships, which is described by partial dependence below.

**FIGURE 3:**
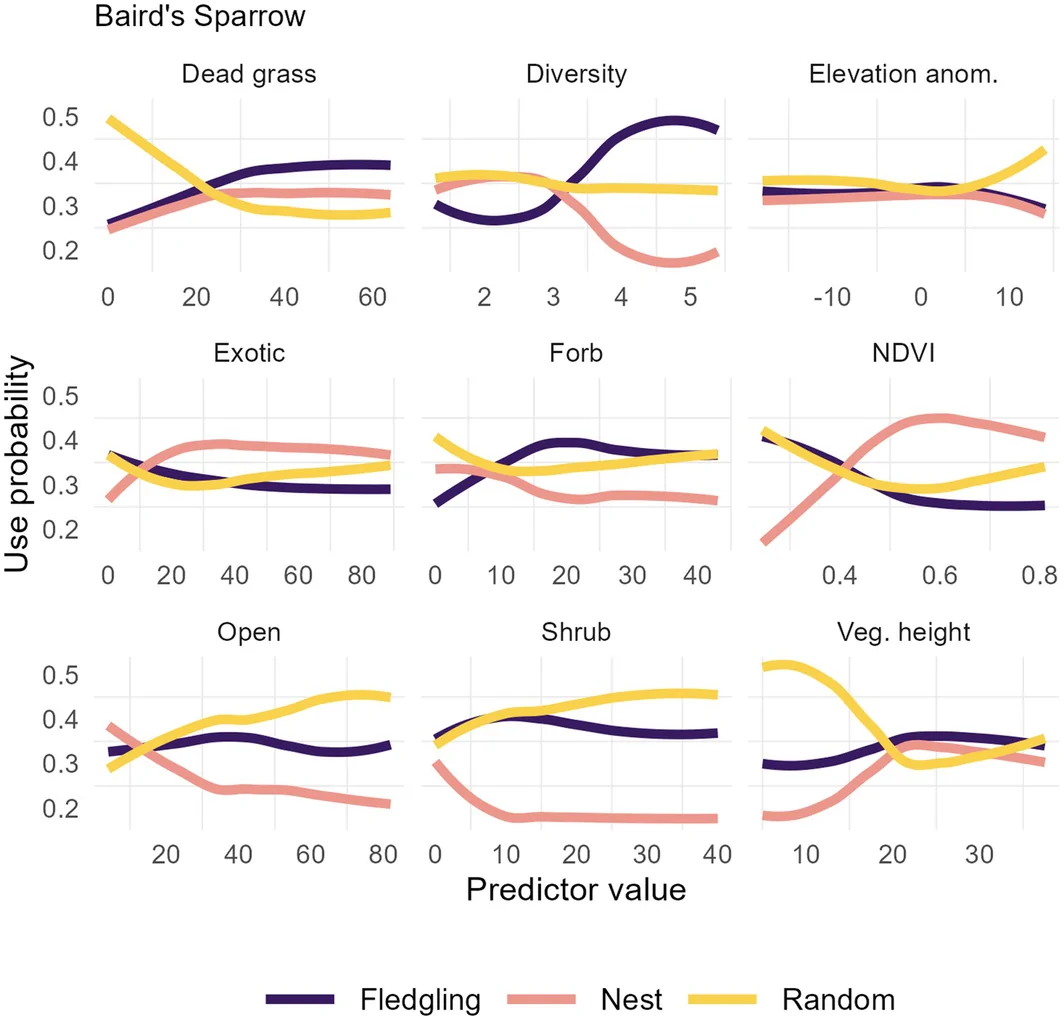
Loess‐smoothed partial dependence displaying directional relationships between habitat covariates and classification probability of Baird's Sparrow fledglings, nest sites, and random locations predicted by multiclass Random Forest. Dead grass, exotic, forb, open, and shrub are shown in units of percent cover; diversity and NDVI are shown as unitless indices; elevation anomaly is shown in meters; and vegetation height is shown in centimeters.

**FIGURE 4:**
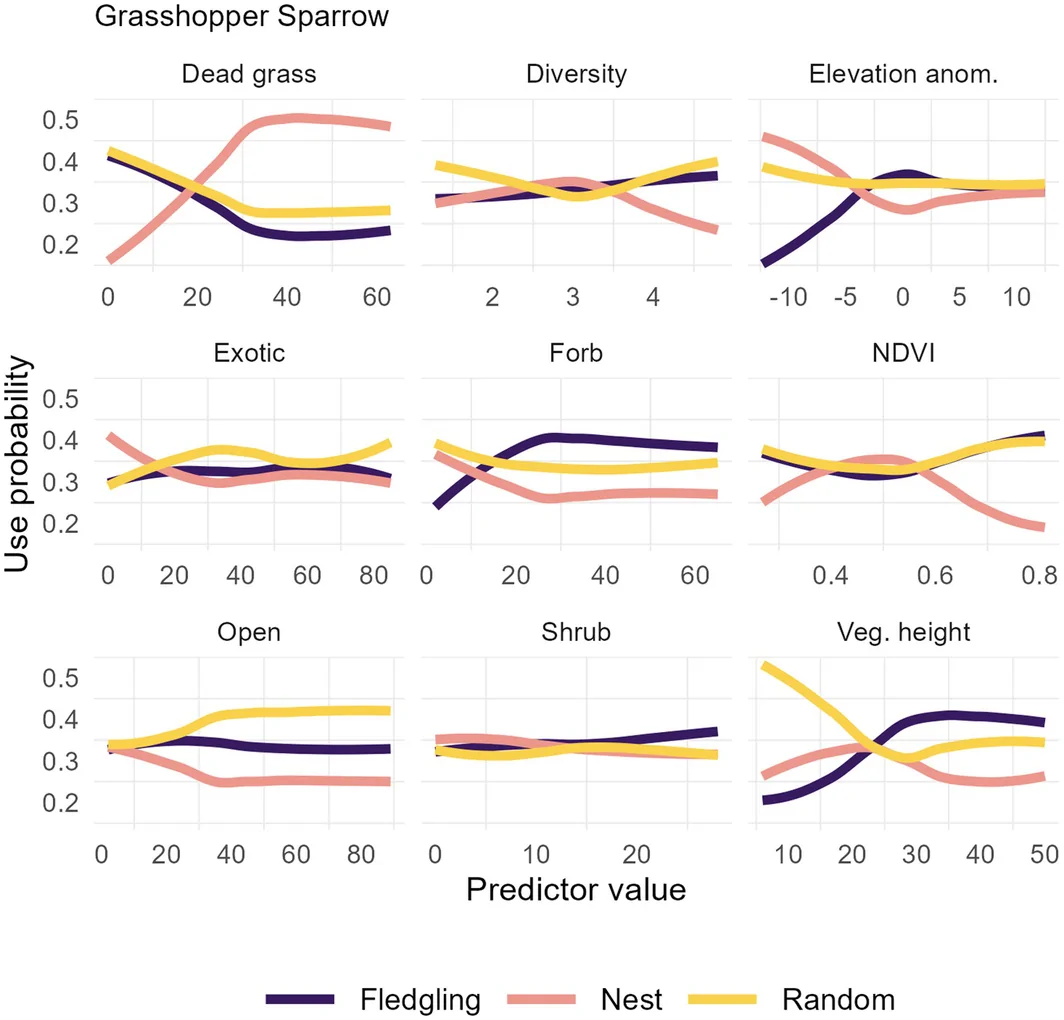
Loess‐smoothed partial dependence displaying directional relationships between habitat covariates and classification probability of Grasshopper Sparrow fledglings, nest sites, and random locations predicted by multiclass Random Forest. Dead grass, exotic, forb, open, and shrub are shown in units of percent cover, diversity and NDVI are shown as unitless indices, elevation anomaly is shown in meters, and vegetation height is shown in centimeters.

Partial dependence predicted that use probability by fledgling Baird's Sparrow decreased with greater NDVI (e.g., primary productivity) relative to random locations while nest sites displayed the opposite relationship (Figure 3). Similarly, Baird's Sparrow fledglings selected for greater cover diversity relative to both conspecific nest sites and random locations (Figure 3). Fledgling Baird's Sparrow were also more associated with forb cover and open cover, and less associated with exotic cover than nests sites (Figure 3). Both Baird's Sparrow fledglings and nesting adults selected for greater vegetation heights and dead grass cover than random locations. Grasshopper Sparrow displayed habitat‐use preferences strongly driven by selection for dead grass cover and lower NDVI values by nesting adults while fledglings used these habitats more similarly to random distributions (Figure 4). Fledgling Grasshopper Sparrow selected for greater forb cover and greater vegetation heights than both nest sites and random locations (Figure 4). Finally, topography also influenced habitat use in Grasshopper Sparrow with fledglings seemingly avoiding local depressions and drainages (Figure 4).

### 3.3 Habitat Use and Fledgling Age

Regressions of habitat use by fledgling age suggested shifting habitat use with age, primarily in Baird's Sparrow (Table S5; Figures S6 and S7). Association of fledgling Baird's Sparrow with bare ground, cover diversity, open cover, NDVI, and shrub cover all increased significantly with age, while use of hill tops (positive elevation anomaly), exotic cover, and live grass all decreased. However, increasing association with NDVI among older fledglings may be partly explained by grassland phenology throughout the season, and NDVI modestly increased as the season progressed (Figure S8). Grasshopper Sparrow displayed fewer and weaker relationships between habitat use and age, but like Baird's Sparrow, older Grasshopper Sparrow fledglings displayed significantly greater association with diverse and open covers, and less association with live grass (Table S5, Figure S7).

## 4 Discussion

We assessed differences in microhabitat selection between post‐fledging and nesting stages into two declining grassland birds, Baird's Sparrow and Grasshopper Sparrow, using multiclass Random Forests (RF). Consistent with existing evidence, we found that Grasshopper Sparrow fledglings selected for taller, live vegetation relative to nest sites and random locations, and fledglings of both species selected greater forb cover. Contrary to our expectation, Baird's Sparrow fledglings also selected more open and diverse cover types than at conspecific nest sites. This pattern may have been driven in part by avoidance of exotic grass cover by Baird's Sparrow fledglings, or possibly ontogenetic shifts in habitat use with progression towards independence. Our findings contribute to existing evidence for differing habitat needs during juvenile life‐stages and illustrate the potential of machine learning (ML) and multiclass models to advance studies of habitat selection. For grassland birds, our work suggests that management efforts to control invasive species and support rangeland heterogeneity, as well as the presence of a forb component, may benefit fledglings.

### 4.1 Habitat Use Across Life History

We found that fledglings of both Baird's Sparrow and Grasshopper Sparrow selected for differing microhabitat characteristics relative to conspecific nest sites and random locations—a finding consistent with existing studies of many taxa (e.g., Desbiez et al. 2020; White et al. 2019). These distinct habitat preferences are likely driven by differing selective pressures acting on adults and juveniles. Adults must select habitats that can balance both survival and fecundity, as well as carry over effects into juvenile life‐history stages (Morris 2025). In birds, nesting adults may attempt to bet‐hedge between habitat characteristics that affect their own survival and lifetime reproductive success, and those that influence number, quality, and survival of offspring—a complicated choice that has made the demonstration of adaptive selection challenging in breeding birds (Chalfoun and Schmidt 2012). However, studies of habitat selection during the post‐fledging phase have revealed a consistent pattern of selection for increased vegetation density and cover across many systems (Jones et al. 2025). This pattern is thought to be driven by low mobility and high mortality during the post‐fledging phase, and the need to seek shelter from predation and exposure (Cox et al. 2014; Jones et al. 2017, 2025; Jones and Bock 2005).

We document support for this pattern in microhabitat selection by fledgling Grasshopper Sparrow, which had greater use probabilities in locations with taller vegetation, higher primary productivity, and greater forb cover relative to conspecific nest sites. These findings align with existing work on post‐fledging habitat selection in the species also reporting greater use of vegetation cover and forbs (Small et al. 2015). Habitat use by fledgling grassland birds is often associated with forb cover (Berkeley et al. 2007; Young et al. 2019), which may provide both concealment and shade, and additional food resources by attracting insects (e.g., Symstad et al. 2000). While Grasshopper Sparrow nest sites showed a strong selection for dead grass cover and avoidance of productive areas relative to random locations, fledglings did not, and even showed marginal avoidance of dead grass. Dead grass is likely selected by adults because it provides suitable concealment for the species' tunneled nests (Vickery 2020)—something we often observed in our study. However, fledglings may choose to leave areas of extensive dead grass because it may impede their movement in early developmental stages, offer fewer food resources, or provide reduced microclimatic buffering (Bernath‐Plaisted et al. 2025). At our study sites, survival of fledgling Grasshopper Sparrow was negatively associated with dead grass (Bernath‐Plaisted et al. 2021), suggesting that more moderate use of this cover type relative to conspecific nest sites is adaptive for fledglings.

Habitat use by fledgling Baird's Sparrow was similar to Grasshopper Sparrow in that fledglings selected for greater forb cover, and although fledgling use locations and nest sites of Baird's Sparrow did not differ in vegetation height, both were associated with greater heights than random locations—likely reflecting the species' association with dense cover (Green et al. 2020; Lipsey and Naugle 2017). Yet, habitat selection by fledgling Baird's Sparrow also presented a contrast to Grasshopper Sparrow. Baird's Sparrow showed distinct selection for greater cover‐class diversity, more use of dead grass, less avoidance of open cover, and less association with NDVI than conspecific nest sites. Below, we discuss several interconnected possible explanations for these findings that illustrate the potential for differing sensitivities among similar species to mediate selection across life history, and the importance of shifts in habitat selection with fledgling development. An important caveat to our findings is that our study was conducted in a single season, and thus future studies that can assess how these relationships may change under different climate and rangeland conditions would likely be valuable.

### 4.2 Species Traits Mediate Selection Patterns

Although Baird's Sparrow fledglings exhibited distinct preferences relative to nest sites and random locations, the directionality of some relationships did not support the hypothesis that fledglings should avoid open cover and nesting adults should select for greater microhabitat diversity (Figure 1). This may have been driven in part by differing sensitivities to non‐native vegetation between the two species. Unlike Grasshopper Sparrow, Baird's Sparrow fledglings responded negatively to exotic vegetation, and displayed modest avoidance of exotic cover relative to nest sites and random locations. Non‐native cover at our sites primarily consisted of cool season grasses that have invaded the Great Plains (Gaskin et al. 2021), and these species often dominated areas of productive vegetation, and sometimes occurred in homogenous monoculture patches (e.g., crested wheatgrass). Thus, favoring the use of native composition may explain Baird's Sparrow fledglings use of more diverse microhabitats relative to homogenous cover provided by invasive grasses. Survival of Baird's Sparrow fledglings at our study sites decreased with exotic cover but increased with vegetation density (Bernath‐Plaisted et al. 2021), indicating competing selective pressures to seek cover, but also to avoid dense vegetation associated with invasive grasses. However, it is important to note that differences in proportion of exotic cover between our Montana and North Dakota study sites could potentially have biased this result, as Baird's Sparrow occurred in greater numbers in Montana. Still, our findings seem to suggest that fledglings used lower proportions of non‐native cover than conspecific nesting adults and available habitat at these sites. This observation is consistent with existing evidence in other specialized grassland birds.

Non‐native cover has been implicated in avoidance and reduced survival in fledglings of other grassland bird species (Fisher and Davis 2011; Young et al. 2019). Although Grasshopper Sparrow and Baird's Sparrow have similar breeding biology in the NGP (Jones et al. 2010; Lipsey and Naugle 2017), Baird's Sparrow has a smaller range and is considered more specialized (Correll et al. 2019). Differences in specialist and generalist traits between the two species may explain greater sensitivity of Baird's Sparrow to invasive species relative to the more widely distributed Grasshopper Sparrow. Differing sensitivities to introduced species among specialists and generalists has been observed across taxa and geography (Clavel et al. 2011; Morrison and Hay 2011), and our study suggests that specialist traits could potentially drive deviation from broadly observed patterns in ontogenetic habitat selection. Given many apparent similarities between Baird's Sparrow and Grasshopper Sparrow, these findings highlight the risks of making management assumptions on this basis. Our work also draws attention to the importance of invasive species management to grassland birds, a major challenge in the Great Plains (e.g., Gaskin et al. 2021), and suggests that more specialized species and juvenile life‐history stages may be uniquely vulnerable to this threat.

### 4.3 Possible Evidence for Shifting Habitat Needs With Fledgling Age

A second factor that may have contributed to unexpected selection for greater cover diversity and lower sensitivity to exposed habitat by fledging Baird's Sparrow could be shifting habitat preferences with development. Differing habitat use between dependent and independent fledglings is often reported in birds (Jones et al. 2025), and specifically in grassland species (Small et al. 2015; Young et al. 2019). This likely occurs as fledglings gain increased mobility, and are driven to seek foraging opportunities when parents cease feeding. Although we did not conduct observation to identify the age of independence in Baird's Sparrow, independence in similar species occurs at ~16–23 days (Small et al. 2015; Young et al. 2019)—overlapping the ages represented in our sample (Figure S5). Regression of habitat use summaries by fledgling age revealed that association of fledgling Baird's Sparrow with open and diverse covers increased with age, while association with vegetation height and live grass decreased. However, somewhat paradoxically, use of greater NDVI values increased for older fledglings. This may suggest that fledglings with greater mobility actively dispersed from areas of non‐native grass at nest sites, and sought other productive vegetation. For example, we observed older Baird's Sparrow fledglings making use of dense vegetation found near ephemeral wetlands and drainages. Evidence of shifting habitat use with age was weaker in the Grasshopper Sparrow, but older fledglings also showed greater association with diverse and open cover.

These observations may represent exploratory foraging behavior by older individuals of both species, and illustrate the importance of different habitat elements with fledgling development. An important limitation of our age‐habitat regressions is that they could not explicitly account for phenological changes in grassland conditions as fledglings aged, and thus it is possible some effects could have been confounded. For example, summary of vegetation at random locations in our sites throughout the season suggested that increased NDVI use with age could also possibly be explained by phenology, though this effect was weak. We saw little evidence of strong seasonal shifts in the other vegetation variables we modeled.

### 4.4 On Multiclass and Machine Learning Approaches to Habitat Selection

Multiclass RF's modeling microhabitat selection by fledglings and nesting adults provided an integrated framework for inference and prediction in both life stages. Although logistic regression has dominated RSF analysis (Northrup et al. 2022), many ecological processes are not binary by nature (Bourel and Segura 2018). In our case, a multiclass approach was natural to the ecological question, and allowed explicit comparison of adult and fledgling selection relative to available habitat rather than separate modeling efforts to address different questions (e.g., nest vs. random, nest vs. fledgling, etc.). An ML framework allowed us to model a large and modestly correlated predictor set without dropping multiple variables, or accepting information loss through dimension reduction (Jia et al. 2022). It also allowed implicit modeling of interactions and nonlinear relationships, which are likely prevalent in many habitat data sets. RF models were successful in making predictions on noisy and class imbalanced data in which predictor space for classes overlapped substantially (Table S3; Figure S4). We suggest that both ML and multiclass approaches have been underutilized in ecological habitat studies (Bourel and Segura 2018; Griffin et al. 2021), and may have potential to improve RSF analyses that examine multiple life stages or seek to directly compare habitat use among multiple species.

## 5 Conclusion

We developed multiclass models of habitat selection in fledglings and nesting adults of two declining grassland birds, Baird's Sparrow and Grasshopper Sparrow, to better understand the importance of differing habitat needs among life‐history stages in these species. We found that, consistent with studies of ontogeny across taxa, fledglings demonstrated distinct habitat use from conspecific nesting adults. Our findings highlight the importance of differing habitat needs and sensitivities across life history in grasslands birds. Structural heterogeneity has broadly become the dominant paradigm in rangeland management and health to benefit a diverse suite of grassland birds in the Great Plains (e.g., Bernath‐Plaisted, Correll, et al. 2023; Derner et al. 2009; Fuhlendorf et al. 2006). However, microhabitat diversity may also be important to meet habitat needs in different ontogenetic stages. Like existing studies of post‐fledging habitat selection in grassland birds, our work indicates that the presence of tall native vegetation and forb cover may benefit younger fledglings seeking concealment and thermoregulation. Yet, our findings also suggest older fledglings likely explore diverse habitat structures and seek foraging opportunity in sparser cover as well. Additionally, our study contributes to a growing body of evidence implicating non‐native vegetation in avoidance by grassland birds, and suggests that fledglings and more specialized species may be espcially sensitive to this threat.

## Author Contributions

**Jacy S. Bernath‐Plaisted:** conceptualization (lead), data curation (lead), formal analysis (lead), funding acquisition (supporting), investigation (lead), methodology (lead), project administration (equal), supervision (equal), visualization (lead), writing – original draft (lead), writing – review and editing (equal). **Nicole A. Sanders:** conceptualization (lead), data curation (lead), formal analysis (supporting), investigation (lead), methodology (supporting), supervision (supporting), writing – original draft (supporting), writing – review and editing (equal). **Katharine J. Ruskin:** conceptualization (supporting), investigation (supporting), methodology (supporting), supervision (supporting), writing – review and editing (equal). **Brian J. Olsen:** conceptualization (supporting), investigation (supporting), methodology (supporting), writing – review and editing (equal). **Arvind O. Panjabi:** conceptualization (supporting), funding acquisition (lead), investigation (supporting), methodology (supporting), supervision (supporting), writing – review and editing (equal). **Maureen D. Correll:** conceptualization (lead), data curation (supporting), funding acquisition (lead), investigation (supporting), methodology (supporting), project administration (equal), supervision (equal), writing – review and editing (equal).

## Funding

This work was supported by the Bobolink Foundation, Conoco Phillips, Montana Fish, Wildlife, and Parks (16‐615), the National Fish and Wildlife Foundation (49599 and 54873), North Dakota Game and Fish Department (15.634), the North Dakota Natural Resources Trust, the Northern Great Plains Joint Venture, the Prairie Potholes Joint Venture, and U.S. Fish and Wildlife Service.

## Conflicts of Interest

The authors declare no conflicts of interest.

## Supporting information


**Table S1:** Class sample size for locations used in multiclass Random Forest classifiers modeling habitat‐selection by fledglings and adult nest sites in two species of North American grassland birds, Baird's Sparrow (BAIS) and Grasshopper Sparrow (GRSP). These data were collected in eastern Montana and western North Dakota, USA, in the summer of 2018.
**Table S2:** Hyperparameters and settings used in multiclass Random Forest classifiers modeling habitat selection by fledglings and adult nest sites in two species of North American grassland birds, Baird's Sparrow (BAIS) and Grasshopper Sparrow (GRSP).
**Table S3:** Mean, standard deviation (SD), maximum value (max), and minimum value (min) at Baird's Sparrow (BAIS) and Grasshopper Sparrow (GRSP) fledgling‐use locations (*n* = 43; *n* = 31), adult nest sites (*n* = 45; *n* = 75), and associated random locations (*n* = 418; *n* = 472), for 12 measured and derived habitat characteristics at grassland study sites in eastern Montana and western North Dakota, USA, in the summer of 2018.
**Table S4:** Performance metrics for multiclass Random Forest classifiers modeling habitat‐selection by fledglings and adult nest sites in two species of North American grassland birds, Baird's Sparrow (BAIS) and Grasshopper Sparrow (GRSP).
**Table S5:** Model output from linear regression of fledgling habitat‐use with age in two species of North American grassland birds, Baird's Sparrow (BAIS) and Grasshopper Sparrow (GRSP), for 12 measured and derived habitat characteristics. These data were collected in eastern Montana and western North Dakota, USA, in the summer of 2018. Effect sizes are scaled for comparison.
**Figure S1:** The breeding ranges of Baird's Sparrow and Grasshopper Sparrow shown in the Northern Great Plains along in study sites where nesting and fledgling habitat data were collected in the summer of 2018. Note that the Grasshopper Sparrow range does not reflect resident sub‐species in the southeastern United States.
**Figure S2:** Normalized density distributions by study site of 12 measured and derived environmental covariates considered in multiclass Random Forests modeling habitat‐selection by two species of North American grassland birds, Baird's Sparrow and Grasshopper Sparrow. These data were collected in eastern Montana and western North Dakota, USA, in the summer of 2018. Distributions include all fledgling‐use locations (*n* = 380), nest sites (*n* = 120), and random locations (*n* = 890) for both species combined.
**Figure S3:** Pearson's correlations for a set of 13 habitat covariates considered for use in modeling of multiclass habitat‐selection of fledglings and adult nest sites in two species of North American grassland birds, Baird's Sparrow and Grasshopper Sparrow. These data were collected at demographic monitoring sites in eastern Montana and western North Dakota, USA in the summer of 2018.
**Figure S4:** Distributions of 12 measured and derived habitat characteristics at fledgling‐use locations, adult nest sites, and random locations for two species of North American grassland birds, Baird's Sparrow (BAIS) and Grasshopper Sparrow (GRSP). These data were collected in eastern Montana and western North Dakota, USA, in the summer of 2018.
**Figure S5:** Sample‐sizes by age in days for fledglings monitored in a habitat study of two species of North American grassland birds, Baird's Sparrow (BAIS) and Grasshopper Sparrow (GRSP). Fledglings were monitored using radio telemetry at study sites in eastern Montana and western North Dakota, USA, in the summer of 2018.
**Figure S6:** Loess‐smoothed trends with 95% confidence intervals displaying fledgling habitat‐use with age in Baird's Sparrow for 12 measured and derived habitat characteristics. These data were collected in eastern Montana and western North Dakota, USA, in the summer of 2018.
**Figure S7:** Loess‐smoothed trends with 95% confidence intervals displaying fledgling habitat‐use with age in Grasshopper Sparrow for 12 measured and derived habitat characteristics. These data were collected in eastern Montana and western North Dakota, USA, in the summer of 2018.
**Figure S8:** Loess‐smoothed trends with 95% confidence intervals displaying vegetation conditions at random locations throughout the season for 12 measured and derived habitat characteristics. These data were collected in eastern Montana and western North Dakota, USA, in the summer of 2018.

## Data Availability

The data and code supporting the findings discussed herein are publically available via permenant respository accessed by the following link: https://doi.org/10.6084/m9.figshare.31685176 https://figshare.com/s/67b9ba85ff939bb3926.

## References

[ece374218-bib-0001] Anders, A. D. , D. C. Dearborn , J. Faaborg , and F. R. T. Iii . 1997. “Juvenile Survival in a Population of Neotropical Migrant Birds.” Conservation Biology 11, no. 3: 698–707. 10.1046/j.1523-1739.1997.95526.x.

[ece374218-bib-0002] Anders, A. D. , and M. R. Marshall . 2005. “Increasing the Accuracy of Productivity and Survival Estimates in Assessing Landbird Population Status.” Conservation Biology 19, no. 1: 66–74. 10.1111/j.1523-1739.2005.00543.x.

[ece374218-bib-0003] Anusha, Y. , R. Visalakshi , and K. Srinivas . 2023. “Imbalanced Data Classification Using Improved Synthetic Minority Over‐Sampling Technique.” Multiagent and Grid Systems 19, no. 2: 117–131. 10.3233/MGS-230007.

[ece374218-bib-0004] Augustine, D. , A. Davidson , K. Dickinson , and B. Van Pelt . 2021. “Thinking Like a Grassland: Challenges and Opportunities for Biodiversity Conservation in the Great Plains of North America.” Rangeland Ecology & Management, Great Plains 78: 281–295. 10.1016/j.rama.2019.09.001.

[ece374218-bib-0005] Berkeley, L. I. , J. P. McCarty , and L. L. Wolfenbarger . 2007. “Postfledging Survival and Movement in Dickcissels ( *Spiza americana* ): Implications for Habitat Management and Conservation.” Auk 124, no. 2: 396–409.

[ece374218-bib-0006] Bernath‐Plaisted, J. S. , M. D. Correll , S. G. Somershoe , et al. 2023. “Review of Conservation Challenges and Possible Solutions for Grassland Birds of the North American Great Plains.” Rangeland Ecology & Management 90: 165–185. 10.1016/j.rama.2023.07.002.

[ece374218-bib-0007] Bernath‐Plaisted, J. S. , A. Panjabi , N. Guido , et al. 2021. “Quantifying Multiple Breeding Vital Rates in Two Declining Grassland Songbirds.” Avian Conservation and Ecology 16, no. 1: 1. 10.5751/ACE-01875-160119.

[ece374218-bib-0008] Bernath‐Plaisted, J. S. , C. A. Ribic , W. B. Hills , P. A. Townsend , and B. Zuckerberg . 2023. “Microclimate Complexity in Temperate Grasslands: Implications for Conservation and Management Under Climate Change.” Environmental Research Letters 18, no. 6: 064023. 10.1088/1748-9326/acd4d3.

[ece374218-bib-0009] Bernath‐Plaisted, J. S. , C. A. Ribic , and B. Zuckerberg . 2025. “Sweating the Small Stuff: Microclimatic Exposure and Species Habitat Associations Inform Climate Vulnerability in a Grassland Songbird Community.” Biology Letters 21, no. 1: 20240599. 10.1098/rsbl.2024.0599. 39837494 PMC11750397

[ece374218-bib-0010] Bourel, M. , and A. M. Segura . 2018. “Multiclass Classification Methods in Ecology.” Ecological Indicators 85: 1012–1021. 10.1016/j.ecolind.2017.11.031.

[ece374218-bib-0011] Breiman, L. 2001. “Random Forests.” Machine Learning 45, no. 1: 5–32. 10.1023/A:1010933404324.

[ece374218-bib-0012] Brussee, B. E. , P. S. Coates , S. T. O'Neil , et al. 2023. “Influence of Fine‐Scale Habitat Characteristics on Sage‐Grouse Nest Site Selection and Nest Survival Varies by Mesic and Xeric Site Conditions.” Ornithological Applications 125, no. 1: duac052. 10.1093/ornithapp/duac052.

[ece374218-bib-0013] Chalfoun, A. D. , and K. A. Schmidt . 2012. “Adaptive Breeding‐Habitat Selection: Is It for the Birds?” Auk 129, no. 4: 589–599. 10.1525/auk.2012.129.4.589.

[ece374218-bib-0014] Chawla, N. V. , K. W. Bowyer , L. O. Hall , and W. P. Kegelmeyer . 2002. “SMOTE: Synthetic Minority Over‐Sampling Technique.” Journal of Artificial Intelligence Research 16: 321–357. 10.1613/jair.953.

[ece374218-bib-0015] Ciotti, B. J. , E. J. Brown , F. Colloca , et al. 2025. “Measuring Juvenile Habitat Quality for Fishes and Invertebrates.” Biological Reviews 100, no. 6: 2346–2395. 10.1111/brv.70050. 40739607 PMC12586312

[ece374218-bib-0016] Clavel, J. , R. Julliard , and V. Devictor . 2011. “Worldwide Decline of Specialist Species: Toward a Global Functional Homogenization?” Frontiers in Ecology and the Environment 9, no. 4: 222–228. 10.1890/080216.

[ece374218-bib-0017] Correll, M. D. , E. H. Strasser , A. W. Green , and A. O. Panjabi . 2019. “Quantifying Specialist Avifaunal Decline in Grassland Birds of the Northern Great Plains.” Ecosphere 10, no. 1: e02523.

[ece374218-bib-0018] Cox, W. A. , F. R. Thompson , A. S. Cox , and J. Faaborg . 2014. “Post‐Fledging Survival in Passerine Birds and the Value of Post‐Fledging Studies to Conservation.” Journal of Wildlife Management 78, no. 2: 183–193.

[ece374218-bib-0019] Derner, J. D. , W. K. Lauenroth , P. Stapp , and D. J. Augustine . 2009. “Livestock as Ecosystem Engineers for Grassland Bird Habitat in the Western Great Plains of North America.” Rangeland Ecology & Management 62, no. 2: 111–118.

[ece374218-bib-0020] Desbiez, A. L. J. , D. Kluyber , G. F. Massocato , L. G. R. Oliveira‐Santos , and N. Attias . 2020. “Life Stage, Sex, and Behavior Shape Habitat Selection and Influence Conservation Strategies for a Threatened Fossorial Mammal.” Italian Journal of Mammalogy 31: 123.

[ece374218-bib-0021] Dorsey, S. S. , D. H. Catlin , S. J. Ritter , et al. 2025. “The Importance of Viewshed in Nest Site Selection of a Ground‐Nesting Shorebird.” PLoS One 20, no. 2: e0319021. 10.1371/journal.pone.0319021. 39977424 PMC11841884

[ece374218-bib-0022] Fisher, R. J. , and S. K. Davis . 2010. “From Wiens to Robel: A Review of Grassland‐Bird Habitat Selection.” Journal of Wildlife Management 74, no. 2: 265–273.

[ece374218-bib-0023] Fisher, R. J. , and S. K. Davis . 2011. “Post‐Fledging Dispersal, Habitat Use, and Survival of Sprague's Pipits: Are Planted Grasslands a Good Substitute for Native?” Biological Conservation 144, no. 1: 263–271.

[ece374218-bib-0024] Fiss, C. J. , D. J. McNeil , A. D. Rodewald , D. Heggenstaller , and J. L. Larkin . 2021. “Cross‐Scale Habitat Selection Reveals Within‐Stand Structural Requirements for Fledgling Golden‐Winged Warblers.” Avian Conservation and Ecology 16, no. 1: art16. 10.5751/ACE-01807-160116.

[ece374218-bib-0025] Frey, C. M. , W. E. Jensen , and K. A. With . 2008. “Topographic Patterns of Nest Placement and Habitat Quality for Grassland Birds in Tallgrass Prairie.” American Midland Naturalist 160, no. 1: 220–234.

[ece374218-bib-0026] Fuhlendorf, S. D. , W. C. Harrell , D. M. Engle , R. G. Hamilton , C. A. Davis , and D. M. Leslie Jr. 2006. “Should Heterogeneity Be the Basis for Conservation? Grassland Bird Response to Fire and Grazing.” Ecological Applications 16, no. 5: 1706–1716. 17069365 10.1890/1051-0761(2006)016[1706:shbtbf]2.0.co;2

[ece374218-bib-0027] Gaskin, J. F. , E. Espeland , C. D. Johnson , et al. 2021. “Managing Invasive Plants on Great Plains Grasslands: A Discussion of Current Challenges.” Rangeland Ecology & Management 78, no. 1: 235–249. 10.1016/j.rama.2020.04.003.

[ece374218-bib-0028] Ghazal, M. , Y. A. Khalil , and H. Hajjdiab . 2015. UAV‐Based Remote Sensing for Vegetation Cover Estimation Using NDVI Imagery and Level Sets Method, 332–337. IEEE International Symposium on Signal Processing and Information Technology (ISSPIT). 10.1109/ISSPIT.2015.7394354.

[ece374218-bib-0029] Green, A. W. , D. C. Pavlacky Jr. , and T. L. George . 2019. “A Dynamic Multi‐Scale Occupancy Model to Estimate Temporal Dynamics and Hierarchical Habitat Use for Nomadic Species.” Ecology and Evolution 9, no. 2: 793–803. 30766669 10.1002/ece3.4822 PMC6362800

[ece374218-bib-0030] Green, M. T. , P. E. Lowther , S. L. Jones , S. K. Davis , and B. C. Dale . 2020. “Baird's Sparrow (Centronyx Bairdii), Version 1.0. *Birds of the World*.” 10.2173/bow.baispa.01.

[ece374218-bib-0031] Greenwell, B. M. 2017. “pdp: An R Package for Constructing Partial Dependence Plots.” R Journal 9, no. 1: 421. 10.32614/RJ-2017-016.

[ece374218-bib-0032] Griffin, L. P. , G. A. Casselberry , K. M. Hart , et al. 2021. “A Novel Framework to Predict Relative Habitat Selection in Aquatic Systems: Applying Machine Learning and Resource Selection Functions to Acoustic Telemetry Data From Multiple Shark Species.” Frontiers in Marine Science 8: 631262. 10.3389/fmars.2021.631262.

[ece374218-bib-0033] Groff, L. A. , A. J. K. Calhoun , and C. S. Loftin . 2017. “Amphibian Terrestrial Habitat Selection and Movement Patterns Vary With Annual Life‐History Period.” Canadian Journal of Zoology 95, no. 6: 433–442. 10.1139/cjz-2016-0148.

[ece374218-bib-0034] Guo, F. , J. J. Buler , J. A. Smolinsky , and D. S. Wilcove . 2023. “Autumn Stopover Hotspots and Multiscale Habitat Associations of Migratory Landbirds in the Eastern United States.” Proceedings of the National Academy of Sciences of the United States of America 120, no. 3: e2203511120. 10.1073/pnas.2203511120. 36623186 PMC9934294

[ece374218-bib-0035] Hanberry, B. B. 2024. “Practical Guide for Retaining Correlated Climate Variables and Unthinned Samples in Species Distribution Modeling, Using Random Forests.” Ecological Informatics 79: 102406. 10.1016/j.ecoinf.2023.102406.

[ece374218-bib-0036] Hannon, S. J. , and K. Martin . 2006. “Ecology of Juvenile Grouse During the Transition to Adulthood.” Journal of Zoology 269, no. 4: 422–433. 10.1111/j.1469-7998.2006.00159.x.

[ece374218-bib-0037] He, L. , R. A. Levine , J. Fan , J. Beemer , and J. Stronach . 2018. “Random Forest as a Predictive Analytics Alternative to Regression in Institutional Research.” Practical Assessment, Research & Evaluation 23, no. 1.

[ece374218-bib-0038] Hvitfeldt, E. 2025. “_themis: Extra Recipe Steps for Dealing With Unblanaced Data (Version R Package Version 1.0.3) [Computer Software].” https://github.com/tidymodels/themis.

[ece374218-bib-0039] Indermaur, L. , and B. R. Schmidt . 2011. “Quantitative Recommendations for Amphibian Terrestrial Habitat Conservation Derived From Habitat Selection Behavior.” Ecological Applications 21, no. 7: 2548–2554. 10.1890/10-2047.1. 22073643

[ece374218-bib-0040] Jaureguiberry, P. , N. Titeux , M. Wiemers , et al. 2022. “The Direct Drivers of Recent Global Anthropogenic Biodiversity Loss.” Science Advances 8, no. 45: eabm9982. 10.1126/sciadv.abm9982. 36351024 PMC9645725

[ece374218-bib-0041] Jedlikowski, J. , P. Chibowski , T. Karasek , and M. Brambilla . 2016. “Multi‐Scale Habitat Selection in Highly Territorial Bird Species: Exploring the Contribution of Nest, Territory and Landscape Levels to Site Choice in Breeding Rallids (Aves: Rallidae).” Acta Oecologica 73: 10–20. 10.1016/j.actao.2016.02.003.

[ece374218-bib-0042] Jia, W. , M. Sun , J. Lian , and S. Hou . 2022. “Feature Dimensionality Reduction: A Review.” Complex & Intelligent Systems 8, no. 3: 2663–2693. 10.1007/s40747-021-00637-x.

[ece374218-bib-0043] Jones, S. L. , J. S. Dieni , and P. J. Gouse . 2010. “Reproductive Biology of a Grassland Songbird Community in Northcentral Montana.” Wilson Journal of Ornithology 122, no. 3: 455–464. 10.1676/08-171.1.

[ece374218-bib-0044] Jones, T. M. , J. D. Brawn , and M. P. Ward . 2017. “Post‐Fledging Habitat Use in the Dickcissel.” Condor 119, no. 3: 497–504.

[ece374218-bib-0045] Jones, T. M. , S. A. Kaiser , and T. S. Sillett . 2025. “Post‐Fledging Ecology of Birds: Emergent Patterns, Knowledge Gaps, and Future Frontiers.” Biological Reviews 101, no. 1: 237–267. 10.1111/brv.70080. 40970343

[ece374218-bib-0046] Jones, Z. F. , and C. E. Bock . 2005. “The Botteri's Sparrow and Exotic Arizona Grasslands: An Ecological Trap or Habitat Regained?” Condor 107, no. 4: 731–741.

[ece374218-bib-0047] Kopitar, L. , L. Cilar , P. Kocbek , and G. Stiglic . 2019. “Local vs. Global Interpretability of Machine Learning Models in Type 2 Diabetes Mellitus Screening.” In Artificial Intelligence in Medicine: Knowledge Representation and Transparent and Explainable Systems, edited by M. Marcos , J. M. Juarez , R. Lenz , et al., 108–119. Springer International Publishing. 10.1007/978-3-030-37446-4_9.

[ece374218-bib-0048] Kramer, A. , G. M. Jones , S. A. Whitmore , et al. 2021. “California Spotted Owl Habitat Selection in a Fire‐Managed Landscape Suggests Conservation Benefit of Restoring Historical Fire Regimes.” Forest Ecology and Management 479: 118576. 10.1016/j.foreco.2020.118576.

[ece374218-bib-0049] Kršák, B. , P. Blišťan , A. Pauliková , et al. 2016. “Use of Low‐Cost UAV Photogrammetry to Analyze the Accuracy of a Digital Elevation Model in a Case Study.” Measurement 91: 276–287. 10.1016/j.measurement.2016.05.028.

[ece374218-bib-0050] Lark, T. J. 2020. “Protecting Our Prairies: Research and Policy Actions for Conserving America's Grasslands.” Land Use Policy 97: 104727. 10.1016/j.landusepol.2020.104727.

[ece374218-bib-0051] Ling, C. X. , J. Huang , and H. Zhang . 2003. “AUC: A Better Measure Than Accuracy in Comparing Learning Algorithms.” In Advances in Artificial Intelligence, edited by Y. Xiang and B. Chaib‐draa , 329–341. Springer. 10.1007/3-540-44886-1_25.

[ece374218-bib-0052] Lipsey, M. K. , and D. E. Naugle . 2017. “Precipitation and Soil Productivity Explain Effects of Grazing on Grassland Songbirds.” Rangeland Ecology & Management 70, no. 3: 331–340.

[ece374218-bib-0053] Marchetti, K. , and T. Price . 1989. “Differences in the Foraging of Juvenile and Adult Birds: The Importance of Developmental Constraints.” Biological Reviews 64, no. 1: 51–70. 10.1111/j.1469-185X.1989.tb00638.x.

[ece374218-bib-0054] Martin, T. E. 2001. “Abiotic vs. Biotic Influences on Habitat Selection of Coexisting Species: Climate Change Impacts?” Ecology 82, no. 1: 175–188.

[ece374218-bib-0055] Martin, T. E. , J. C. Oteyza , A. J. Boyce , P. Lloyd , and R. Ton . 2015. “Adult Mortality Probability and Nest Predation Rates Explain Parental Effort in Warming Eggs With Consequences for Embryonic Development Time.” American Naturalist 186, no. 2: 223–236. 10.1086/681986. 26655151

[ece374218-bib-0056] Molnar, C. 2022. Interpretable Machine Learning: A Guide for Making Black Box Models Explainable. 2nd ed. https://christophm.github.io/interpretable‐ml‐book/.

[ece374218-bib-0057] Morris, D. W. 2025. “Habitat‐Dependent Life History.” Canadian Journal of Fisheries and Aquatic Sciences 82: 1–14. 10.1139/cjfas-2024-0256.

[ece374218-bib-0058] Morrison, W. E. , and M. E. Hay . 2011. “Herbivore Preference for Native vs. Exotic Plants: Generalist Herbivores From Multiple Continents Prefer Exotic Plants That Are Evolutionarily naïve.” PLoS One 6, no. 3: e17227. 10.1371/journal.pone.0017227. 21394202 PMC3048865

[ece374218-bib-0059] Northrup, J. M. , M. B. Hooten , C. R. Anderson Jr. , and G. Wittemyer . 2013. “Practical Guidance on Characterizing Availability in Resource Selection Functions Under a Use–Availability Design.” Ecology 94, no. 7: 1456–1463. 10.1890/12-1688.1. 23951705

[ece374218-bib-0060] Northrup, J. M. , E. Vander Wal , M. Bonar , et al. 2022. “Conceptual and Methodological Advances in Habitat‐Selection Modeling: Guidelines for Ecology and Evolution.” Ecological Applications 32, no. 1: e02470. 10.1002/eap.2470. 34626518 PMC9285351

[ece374218-bib-0061] Pichler, M. , and F. Hartig . 2023. “Machine Learning and Deep Learning—A Review for Ecologists.” Methods in Ecology and Evolution 14, no. 4: 994–1016. 10.1111/2041-210X.14061.

[ece374218-bib-0062] Pitkänen, S. 1998. “The Use of Diversity Indices to Assess the Diversity of Vegetation in Managed Boreal Forests.” Forest Ecology and Management 112, no. 1: 121–137. 10.1016/S0378-1127(98)00319-3.

[ece374218-bib-0063] R Core Team . 2026. R: A Language for Statistical Computing [Computer Software]. R Foundation for statistical computing. https://www.R‐project.org/.

[ece374218-bib-0064] Rappole, J. H. , and A. R. Tipton . 1991. “New Harness Design for Attachment of Radio Transmitters to Small Passerines.” Journal of Field Ornithology 62, no. 3: 335–337.

[ece374218-bib-0065] Raybuck, D. W. , J. L. Larkin , S. H. Stoleson , and T. J. Boves . 2020. “Radio‐Tracking Reveals Insight Into Survival and Dynamic Habitat Selection of Fledgling Cerulean Warblers.” Condor: Ornithological Applications 122, no. 1: duz063. 10.1093/condor/duz063.

[ece374218-bib-0066] Robin, X. , N. Turck , A. Hainard , et al. 2011. “pROC: An Open‐Source Package for R and S+ to Analyze and Compare ROC Curves.” BMC Bioinformatics 12, no. 1: 77. 10.1186/1471-2105-12-77. 21414208 PMC3068975

[ece374218-bib-0067] Rodewald, A. D. 2004. “Nest‐Searching Cues and Studies of Nest‐Site Selection and Nesting Success.” Journal of Field Ornithology 75, no. 1: 31–39.

[ece374218-bib-0068] Rosenberg, K. V. , A. M. Dokter , P. J. Blancher , et al. 2019. “Decline of the North American Avifauna.” Science 366, no. 6461: 120–124. 31604313 10.1126/science.aaw1313

[ece374218-bib-0069] Russell, E. M. 2001. “Avian Life Histories: Is Extended Parental Care the Southern Secret?” Emu 100, no. 5: 377–399. 10.1071/MU0005S.

[ece374218-bib-0070] Segura, L. N. , D. A. Masson , and M. G. Gantchoff . 2012. “Microhabitat Nest Cover Effect on Nest Survival of the Red‐Crested Cardinal.” Wilson Journal of Ornithology 124, no. 3: 506–512. 10.1676/11-181.1.

[ece374218-bib-0071] Shew, J. J. , C. K. Nielsen , and D. W. Sparling . 2019. “Finer‐Scale Habitat Predicts Nest Survival in Grassland Birds More Than Management and Landscape: A Multi‐Scale Perspective.” Journal of Applied Ecology 56, no. 4: 929–945. 10.1111/1365-2664.13317.

[ece374218-bib-0072] Sidumo, B. , E. Sonono , and I. Takaidza . 2022. “An Approach to Multi‐Class Imbalanced Problem in Ecology Using Machine Learning.” Ecological Informatics 71: 101822. 10.1016/j.ecoinf.2022.101822.

[ece374218-bib-0073] Small, D. M. , P. J. Blank , and B. Lohr . 2015. “Habitat Use and Movement Patterns by Dependent and Independent Juvenile Grasshopper Sparrows During the Post‐Fledging Period.” Journal of Field Ornithology 86, no. 1: 17–26.

[ece374218-bib-0074] Spiller, K. J. , and D. I. King . 2021. “Breeding Habitat Associations of Eastern Whip‐Poor‐Wills in Managed Forests.” Journal of Wildlife Management 85, no. 5: 1009–1016. 10.1002/jwmg.22045.

[ece374218-bib-0075] Symstad, A. J. , E. Siemann , and J. Haarstad . 2000. “An Experimental Test of the Effect of Plant Functional Group Diversity on Arthropod Diversity.” Oikos 89, no. 2: 243–253. 10.1034/j.1600-0706.2000.890204.x.

[ece374218-bib-0076] Tagmann‐Ioset, A. , M. Schaub , T. S. Reichlin , N. Weisshaupt , and R. Arlettaz . 2012. “Bare Ground as a Crucial Habitat Feature for a Rare Terrestrially Foraging Farmland Bird of Central Europe.” Acta Oecologica 39: 25–32. 10.1016/j.actao.2011.11.003.

[ece374218-bib-0077] Thomson, D. L. , S. R. Baillie , and W. J. Peach . 1999. “A Method for Studying Post‐Fledging Survival Rates Using Data From Ringing Recoveries.” Bird Study 46, no. sup1: S104–S111. 10.1080/00063659909477237.

[ece374218-bib-0078] Thorsen, N. H. , J. E. Hansen , O.‐G. Støen , J. Kindberg , A. Zedrosser , and S. C. Frank . 2022. “Movement and Habitat Selection of a Large Carnivore in Response to Human Infrastructure Differs by Life Stage.” Movement Ecology 10, no. 1: 52. 10.1186/s40462-022-00349-y. 36447280 PMC9706841

[ece374218-bib-0079] Vickery, P. D. 2020. “Grasshopper Sparrow ( *Ammodramus savannarum* ), Version 1.0. *Birds of the World*.” 10.2173/bow.graspa.01.

[ece374218-bib-0080] Vitz, A. C. , and A. D. Rodewald . 2011. “Influence of Condition and Habitat Use on Survival of Post‐Fledging Songbirds.” Condor: Ornithological Applications 113, no. 2: 400–411. 10.1525/cond.2011.100023.

[ece374218-bib-0081] White, C. F. , K. Lyons , S. J. Jorgensen , et al. 2019. “Quantifying Habitat Selection and Variability in Habitat Suitability for Juvenile White Sharks.” PLoS One 14, no. 5: e0214642. 10.1371/journal.pone.0214642. 31067227 PMC6505937

[ece374218-bib-0082] Winter, M. , S. E. Hawks , J. A. Shaffer , and D. H. Johnson . 2003. “Guidelines for Finding Nests of Passerine Birds in Tallgrass Prairie.” Prairie Naturalist 35, no. 3: 16.

[ece374218-bib-0083] Wright, M. N. , and A. Ziegler . 2017. “Ranger: A Fast Implementation of Random Forests for High Dimensional Data in C++ and R.” Journal of Statistical Software 77, no. 1: 1–17. 10.18637/jss.v077.i01.

[ece374218-bib-0084] Young, A. C. , W. A. Cox , J. P. McCarty , and L. L. Wolfenbarger . 2019. “Postfledging Habitat Selection and Survival of Henslow's Sparrow: Management Implications for a Critical Life Stage.” Avian Conservation and Ecology 14, no. 2: art10.

