# Supplementary material for: In A Class of Their Own: Multiclass Random Forests Reveal Distinct Habitat Needs of Fledglings Relative to Adults in Two Grassland Birds
Source: Ecol Evol. 2026 Aug 19;16(8):e74218. doi: 10.1002/ece3.74218 (PMC13490722; doi:10.1002/ece3.74218)
Supplement: Supplementary file 1 [file ECE3-16-e74218-s001.docx]

**Supporting information for:**

In a class of their own: multiclass Random Forests reveal distinct habitat needs of fledglings relative to adults in two grassland birds

Jacy S. Bernath-Plaisted^[1]*^, Nicole A. Sanders^2^, Katherine J. Ruskin^2^, Brian J. Olsen^3^, Arvind O. Panjabi^1^, Maureen D. Correll^4^

*^1^Bird Conservancy of the Rockies, 230 Cherry St Suite 150, Fort Collins, CO 80521, U.S.A.*

*^2^Ecology and Environmental Sciences Program, University of Maine, Libby Hall, 48 Grove St Ext Ste 106, Orono, ME 04469*

*^3^School of Biology & Ecology, University of Maine, 5722 Deering Hall, Orono, ME 04469*

*^4^U.S. Fish & Wildlife Service, 300 Westgate Center Drive, Hadley MA 01035*

*Corresponding author: [jacy.bernath-plaisted@birdconservancy.org](mailto:jacy.bernath-plaisted@birdconservancy.org)

**Supporting text**

*UAS flights and imagery*

We only operated UAS in optimal spectral conditions when cloud cover was minimal, winds were low (< 25 kph), and between the hours 10:00 and 14:00 MST to minimize shadowing. We flew missions in systematic grids and collected data with an 80% photo overlap to ensure spatial coverage. UAS altitude during flights never exceeded 120 m (AGL), and UAS flew missions at a speed of ~ 40 kph. Spectral data included bandwidths within the visible light spectrum (red, green, blue) gathered with a Sensor Optimized for Drone Applications (SODA; senseFly, Switzerland), which rendered rasters produced from collected imagery at a resolution of 2-4 cm. We also recorded four spectral bands including visible green, visible red, red edge, and near infrared (ranging from wavelengths 550-790 nanometers) using a Parrot Sequoia (Parrot SA, Paris, France) sensor, which produced rasters from collected imagery at a resolution of 11-15 cm. We used Pix4D imagery processing software, (version 4.1, Pix4D SA, Lausanne, Switzerland) to align georeferenced images (raster images associated with spatial locations), generate point clouds, create orthomosaics and create Digital Surface Models (DSM) from these UAS-collected data. We used a Trimble R2 (Trimble, Sunnyvale, California) to collect ground control points that were later included in the photogrammetry process to correct georeferenced images to sub-decimeter accuracy. For nest sites, we used spectral data collected closest to estimated initiation-date to best represent conditions that were selected for. Initiation dates were estimated by back dating from hatch date or nestling age, and calculating initiation with clutch size and average incubation period.

*Derived habitat variables*

We created an “open” habitat cover class calculated as the sum of the bare ground and litter cover measurements. We did this because we felt the two classes were functionally similar as exposed habitat, and litter cover was low at our sites (Figure S2) and uninformative in initial models. We also created a habitat diversity variable summarizing the diversity of cover classes (including bare ground, dead grass, forb, litter, live grass, and shrub) present at each location surveyed using the inverse Simpson’s diversity index. Such indices have proven effective for quantifying heterogeneity in compositional habitat data (Pitkänen, 1998). We used the *vegan* package (Oksanen et al., 2026) in Program R (R Core Team, 2026) to calculate the index. Finally, we used raw elevation collected by UAS to create an elevation-anomaly variable, calculated as the mean elevation at each study pasture subtracted from each observation elevation. Thus, this variable characterized whether a given location tended to be higher or lower than local conditions at the site (e.g., hilltops and drainages). We did this because mean elevations differed among our four study-pastures and caused elevation observations to cluster into two groups. This resulted in a misleading bimodal distribution in elevation use patterns that did not represent selection (Figure S2).

*Random Forest tuning and hyperparameters*

We tuned models over standard Random Forest hyperparameters including *mtry* (number of variables in first split), *minimum node size* (minimum observations per node), and *sample fraction* (proportion of sample used to train each tree) using the tuneRanger () function from *ranger*. Additionally, we evaluated both models over a range of *n trees* (number of model iterations; 500, 1000, 5000, 10000) and selected a final value based on the point at which performance leveled off with increased trees. Finally, we also tested a range of proportions (60-40, 65-45, 70-30, 75-25, 80-20) for partitioning training and test data. We did this because we did not oversample minority classes in our test data to follow best practices for SMOTE, and model evaluation was sensitive to the number of nests (the lowest frequency class) present in the test data. All hyperparameters and settings used in our final models are available in Table S2.

**References**

Oksanen, J., Simpson, G., Blanchet, F., Kindt, R., Legendre, P., Minchin, P., O’Hara, R., Solymos, P., Stevens, M., Szoecs, E., Wagner, H., Barbour, M., Bedward, M., Bolker, B., Borcard, D., Borman, T., Carvalho, G., De Caceres, M., Durand, S., … Weedon, J. (2026). *vegan: Community Ecology Package* (Version R Package version 2.7-3) [Computer software]. https://vegandevs.github.io/vegan/

Pitkänen, S. (1998). The use of diversity indices to assess the diversity of vegetation in managed boreal forests. *Forest Ecology and Management*, *112*(1), 121–137. https://doi.org/10.1016/S0378-1127(98)00319-3

R Core Team. (2026). *R: A language for statistical computing* [Computer software]. R Foundation for statistical computing. https://www.R-project.org/

**Supporting tables and figures**

**Table S1.** Class sample-size for locations used in multiclass Random Forest classifiers modelling habitat-selection by fledglings and adult nest-sites in two species of North American grassland birds, Baird’s Sparrow (BAIS) and Grasshopper Sparrow (GRSP). These data were collected in eastern Montana and western North Dakota, USA, in the summer of 2018.

| **Species** | **Class** | **Sample (*n*)** |
| --- | --- | --- |
| Baird’s Sparrow | Fledgling use | 170 |
| Baird’s Sparrow | Nest site | 45 |
| Baird’s Sparrow | Random location | 230 |
| Grasshopper Sparrow | Fledgling use | 210 |
| Grasshopper Sparrow | Nest site | 75 |
| Grasshopper Sparrow | Random location | 274 |

**Table S2.** Hyperparameters and settings used in multiclass Random Forest classifiers modelling habitat-selection by fledglings and adult nest-sites in two species of North American grassland birds, Baird’s Sparrow (BAIS) and Grasshopper Sparrow (GRSP).

| **Species** | ***Mtry*** | ***Min. node*** | ***Sample frac.*** | ***n trees*** | **Partition** |
| --- | --- | --- | --- | --- | --- |
| BAIS | 2 | 2 | 0.77 | 1000 | 70-30 |
| GRSP | 2 | 2 | 0.89 | 10,000 | 65-35 |

**Table S3.** Mean, standard deviation (SD), maximum value (max), and minimum value (min) at Baird’s Sparrow (BAIS) and Grasshopper Sparrow (GRSP) fledgling-use locations (*n* = 43; *n* =31), adult nest-sites (*n* = 45; *n* = 75), and associated random locations (*n* = 418; *n* = 472), for 12 measured and derived habitat characteristics at grassland study sites in eastern Montana and western North Dakota, USA, in the summer of 2018.

| **Species** | **Use** | **Variable** | ***Mean*** | | ***SD*** | ***Max*** | ***Min*** |
| --- | --- | --- | --- | --- | --- | --- | --- |
| BAIS | Fledgling | Bare ground | | 20.9 | 18.1 | 78 | 0 |
| BAIS | Nest | Bare ground | | 10.8 | 13.3 | 55 | 0 |
| BAIS | Random | Bare ground | | 26.6 | 21.9 | 81 | 0 |
| GRSP | Fledgling | Bare ground | | 10 | 10.7 | 61 | 0 |
| GRSP | Nest | Bare ground | | 9.9 | 11.9 | 60 | 1 |
| GRSP | Random | Bare ground | | 14.1 | 15.8 | 88 | 0 |
| BAIS | Fledgling | Dead grass | | 25 | 14.2 | 60 | 3 |
| BAIS | Nest | Dead grass | | 25 | 15.7 | 62 | 3 |
| BAIS | Random | Dead grass | | 20.8 | 14.2 | 64 | 0 |
| GRSP | Fledgling | Dead grass | | 12.4 | 10 | 55 | 0 |
| GRSP | Nest | Dead grass | | 22.4 | 12.3 | 63 | 1 |
| GRSP | Random | Dead grass | | 14 | 10.5 | 48 | 0 |
| BAIS | Fledgling | Diversity | | 3.2 | 0.8 | 5.4 | 1.5 |
| BAIS | Nest | Diversity | | 2.7 | 0.6 | 4.1 | 1.3 |
| BAIS | Random | Diversity | | 2.9 | 0.7 | 5.2 | 1.3 |
| GRSP | Fledgling | Diversity | | 2.7 | 0.8 | 4.7 | 1.2 |
| GRSP | Nest | Diversity | | 2.9 | 0.7 | 4.8 | 1.5 |
| GRSP | Random | Diversity | | 2.8 | 0.8 | 5.5 | 1.3 |
| BAIS | Fledgling | Elev. anom. | | 1.8 | 5 | 13.9 | -13.9 |
| BAIS | Nest | Elev. anom. | | 2.5 | 5.5 | 14 | -9.6 |
| BAIS | Random | Elev. anom. | | 2 | 5.6 | 14 | -18 |
| GRSP | Fledgling | Elev. anom. | | -1.2 | 3.5 | 7.9 | -8.7 |
| GRSP | Nest | Elev. anom. | | -1.8 | 5.3 | 11.8 | -11.1 |
| GRSP | Random | Elev. anom. | | -1.8 | 4.1 | 12.5 | -13.4 |
| BAIS | Fledgling | Exotic | | 8.4 | 13.3 | 80 | 0 |
| BAIS | Nest | Exotic | | 24.3 | 27.9 | 91 | 0 |
| BAIS | Random | Exotic | | 11.2 | 19 | 89 | 0 |
| GRSP | Fledgling | Exotic | | 42.7 | 21.4 | 85 | 0 |
| GRSP | Nest | Exotic | | 44.2 | 25 | 85 | 0 |
| GRSP | Random | Exotic | | 43.2 | 21.9 | 90 | 0 |
| BAIS | Fledgling | Forb | | 13.1 | 7.5 | 40 | 1 |
| BAIS | Nest | Forb | | 10.2 | 6.9 | 32 | 2 |
| BAIS | Random | Forb | | 11.7 | 8.4 | 43 | 0 |
| GRSP | Fledgling | Forb | | 19.8 | 13 | 74 | 2 |
| GRSP | Nest | Forb | | 13.2 | 9.1 | 41 | 2 |
| GRSP | Random | Forb | | 16.3 | 11.7 | 67 | 1 |
| BAIS | Fledgling | Litter | | 5.4 | 4.4 | 31 | 1 |
| BAIS | Nest | Litter | | 6.4 | 4.7 | 20 | 1 |
| BAIS | Random | Litter | | 5.1 | 6.3 | 76 | 0 |
| GRSP | Fledgling | Litter | | 5 | 4.1 | 33 | 0 |
| GRSP | Nest | Litter | | 5.6 | 3.4 | 19 | 1 |
| GRSP | Random | Litter | | 5.1 | 4 | 26 | 0 |
| BAIS | Fledgling | Live grass | | 33.2 | 18.8 | 82 | 3 |
| BAIS | Nest | Live grass | | 46.5 | 20.5 | 87 | 14 |
| BAIS | Random | Live grass | | 33.5 | 19.6 | 89 | 3 |
| GRSP | Fledgling | Live grass | | 50.3 | 17.9 | 91 | 5 |
| GRSP | Nest | Live grass | | 46.7 | 15.5 | 80 | 12 |
| GRSP | Random | Live grass | | 48.9 | 18.3 | 88 | 4 |
| BAIS | Fledgling | NDVI | | 0.4 | 0.1 | 0.7 | 0.3 |
| BAIS | Nest | NDVI | | 0.5 | 0.1 | 0.8 | 0.2 |
| BAIS | Random | NDVI | | 0.4 | 0.1 | 0.7 | 0.2 |
| GRSP | Fledgling | NDVI | | 0.6 | 0.1 | 0.8 | 0.2 |
| GRSP | Nest | NDVI | | 0.5 | 0.1 | 0.7 | 0.3 |
| GRSP | Random | NDVI | | 0.5 | 0.1 | 0.8 | 0.3 |
| BAIS | Fledgling | Open | | 26.3 | 18 | 80 | 4 |
| BAIS | Nest | Open | | 17.2 | 13.4 | 56 | 4 |
| BAIS | Random | Open | | 31.6 | 21.4 | 82 | 4 |
| GRSP | Fledgling | Open | | 14.9 | 10.5 | 63 | 2 |
| GRSP | Nest | Open | | 15.5 | 11.3 | 62 | 2 |
| GRSP | Random | Open | | 19.2 | 15.3 | 89 | 2 |
| BAIS | Fledgling | Shrub | | 2.3 | 5.7 | 33 | 0 |
| BAIS | Nest | Shrub | | 0.2 | 0.5 | 2 | 0 |
| BAIS | Random | Shrub | | 2.3 | 6.5 | 40 | 0 |
| GRSP | Fledgling | Shrub | | 2.5 | 7.2 | 60 | 0 |
| GRSP | Nest | Shrub | | 1.3 | 2.5 | 12 | 0 |
| GRSP | Random | Shrub | | 1.6 | 4.2 | 27 | 0 |
| BAIS | Fledgling | Veg. height | | 21.5 | 5.4 | 36 | 9 |
| BAIS | Nest | Veg. height | | 23.8 | 6 | 40 | 11 |
| BAIS | Random | Veg. height | | 19.5 | 6.4 | 41 | 5 |
| GRSP | Fledgling | Veg. height | | 25.5 | 5.7 | 50 | 15 |
| GRSP | Nest | Veg. height | | 22.7 | 5.4 | 36 | 10 |
| GRSP | Random | Veg. height | | 23 | 6 | 42 | 6 |

**Table S4.** Performance metrics for multiclass Random Forest classifiers modelling habitat-selection by fledglings and adult nest-sites in two species of North American grassland birds, Baird’s Sparrow (BAIS) and Grasshopper Sparrow (GRSP).

| **Species** | **Balanced accuracy** | **Sensitivity (fledgling)** | **Sensitivity (nest)** | **Specificity (fledgling)** | **Specificity (nest)** | **AUC** |
| --- | --- | --- | --- | --- | --- | --- |
| BAIS | 70% | 49% | 69% | 72% | 91% | 0.73 |
| GRSP | 63% | 52% | 35% | 74% | 89% | 0.70 |
|  |  |  |  |  |  |  |

**Table S5.** Model output from linear regression of fledgling habitat-use with age in two species of North American grassland birds, Baird’s Sparrow (BAIS) and Grasshopper Sparrow (GRSP), for 12 measured and derived habitat characteristics. These data were collected in eastern Montana and western North Dakota, USA, in the summer of 2018. Effect sizes are scaled for comparison.

| **Species** | **Variable** | ***β*** | **Std. Error** | ***P*-value** | ***R^2^*** |
| --- | --- | --- | --- | --- | --- |
| BAIS | Bare ground | 0.039 | 0.013 | 0.003* | 0.054 |
| BAIS | Dead grass | 0.018 | 0.013 | 0.173 | 0.012 |
| BAIS | Diversity | 0.040 | 0.013 | 0.002* | 0.057 |
| BAIS | Elev. anomaly | -0.063 | 0.012 | 0.000* | 0.144 |
| BAIS | Exotic | -0.046 | 0.013 | 0.000* | 0.076 |
| BAIS | Forb | -0.007 | 0.013 | 0.599 | 0.002 |
| BAIS | Litter | 0.018 | 0.013 | 0.169 | 0.012 |
| BAIS | Live grass | -0.062 | 0.012 | 0.000* | 0.139 |
| BAIS | NDVI | 0.058 | 0.012 | 0.000* | 0.121 |
| BAIS | Open | 0.043 | 0.013 | 0.001* | 0.067 |
| BAIS | Shrub | 0.032 | 0.013 | 0.015* | 0.037 |
| BAIS | Veg. height | -0.047 | 0.013 | 0.000* | 0.08 |
| GRSP | Bare ground | 0.021 | 0.012 | 0.083 | 0.015 |
| GRSP | Dead grass | 0.015 | 0.012 | 0.224 | 0.007 |
| GRSP | Diversity | 0.041 | 0.012 | 0.001* | 0.056 |
| GRSP | Elev. anomaly | -0.034 | 0.012 | 0.005 | 0.039 |
| GRSP | Exotic | -0.002 | 0.012 | 0.858 | 0.000 |
| GRSP | Forb | 0.019 | 0.012 | 0.120 | 0.012 |
| GRSP | Litter | 0.028 | 0.012 | 0.022* | 0.026 |
| GRSP | Live grass | -0.035 | 0.012 | 0.004* | 0.042 |
| GRSP | NDVI | 0.015 | 0.012 | 0.212 | 0.008 |
| GRSP | Open | 0.033 | 0.012 | 0.006* | 0.037 |
| GRSP | Shrub | -0.014 | 0.012 | 0.264 | 0.006 |
| GRSP | Veg. height | -0.005 | 0.012 | 0.698 | 0.001 |

**Figure S1.** The breeding ranges of Baird’s Sparrow and Grasshopper Sparrow shown in the Northern Great Plains along in study sites where nesting and fledgling habitat data were collected in the summer of 2018. Note that the Grasshopper Sparrow range does not reflect resident sub-species in the southeastern United States.

**Figure S2.** Normalized density distributions by study site of 12 measured and derived environmental covariates considered in multiclass Random Forests modeling habitat-selection by two species of North American grassland birds, Baird’s Sparrow and Grasshopper Sparrow. These data were collected in eastern Montana and western North Dakota, USA, in the summer of 2018. Distributions include all fledgling-use locations (*n* = 380), nest sites (*n* = 120), and random locations (*n* = 890) for both species combined.

**Figure S3.** Pearson’s correlations for a set of 13 habitat covariates considered for use in modeling of multiclass habitat-selection of fledglings and adult nest-sites in two species of North American grassland birds, Baird’s Sparrow and Grasshopper Sparrow. These data were collected at demographic monitoring sites in eastern Montana and western North Dakota, USA in the summer of 2018.

**Figure S4.** Distributions of 12 measured and derived habitat characteristics at fledgling-use locations, adult nest-sites, and random locations for two species of North American grassland birds, Baird’s Sparrow (BAIS) and Grasshopper Sparrow (GRSP). These data were collected in eastern Montana and western North Dakota, USA, in the summer of 2018.

**Figure S5.** Sample-sizes by age in days for fledglings monitored in a habitat study of two species of North American grassland birds, Baird’s Sparrow (BAIS) and Grasshopper Sparrow (GRSP). Fledglings were monitored using radio telemetry at study sites in eastern Montana and western North Dakota, USA, in the summer of 2018.

**Figure S6.** Loess-smoothed trends with 95% confidence intervals displaying fledgling habitat-use with age in Baird’s Sparrow for 12 measured and derived habitat characteristics. These data were collected in eastern Montana and western North Dakota, USA, in the summer of 2018.

**Figure S7.** Loess-smoothed trends with 95% confidence intervals displaying fledgling habitat-use with age in Grasshopper Sparrow for 12 measured and derived habitat characteristics. These data were collected in eastern Montana and western North Dakota, USA, in the summer of 2018.

**Figure S8.** Loess-smoothed trends with 95% confidence intervals displaying vegetation conditions at random locations throughout the season for 12 measured and derived habitat characteristics. These data were collected in eastern Montana and western North Dakota, USA, in the summer of 2018.

1. ^*^ Corresponding author: 230 Cherry St Suite 150, Fort Collins, CO 80521. | [jacy.bernath-plaisted@birdconservancy.org](mailto:bernathplais@wisc.edu) | (517) 927-5463
